# Importance of imaging to wound care practice

**DOI:** 10.1111/iwj.14082

**Published:** 2023-01-30

**Authors:** Douglas Queen, Keith Gordon Harding

Medical imaging has greatly advanced health care in the past three decades. Its use has allowed clinicians to identify injuries, conditions, and diseases. From broken bones to compromised organs to cancer detection, early and/or accurate diagnosis can save limbs and lives. Medical imaging offers quicker and more reliable information, and has drastically improved patient outcomes and helped doctors achieve better results. For example, medical imaging guides doctors in surgery for improved precision and accuracy. Imaging permits doctors to assess disease progression or severity of an injury. This information helps doctors choose the right approach and treatments.

What is regarded as medical imaging? The FDA defines it as, “Medical imaging refers to several different technologies that are used to view the human body in order to diagnose, monitor, or treat medical conditions. Each type of technology gives different information about the area of the body being studied or treated, related to possible disease, injury, or the effectiveness of medical treatment.”

Imaging is familiar to most clinicians regarding X‐rays, ultrasound, and MRI as examples. Some are becoming familiar to those providing wound care. Imaging has become an essential part of the clinical record for wound care patients by using photography.^1^ However, the area of imaging technology is just evolving within wound care, providing more new diagnostic tools,^2^ some of which will be highlighted below.

Medical imaging has been an important tool for many years throughout all aspects of medicine. Historically, wound care clinicians do not access such technology within wound care centres. This appears to be changing. There are new, small, portable, point‐of‐care devices being developed that can easily fit into the wound care algorithm. The future of wound care^3^ will include the adoption and implementation of these new diagnostic approaches, helping prevent, diagnose, treat, and monitor wound progress.^4^

Wound assessment, for the most part, relies on visual evaluation by clinicians, both expert and non‐expert.^5^ Such assessment is largely subjective and presents the opportunity for development of new technologies to remove subjectivity from the assessment process. Advanced solutions have, therefore, been developed where digital photography, usually using smart phones, is supported by advanced machine learning software. Such approaches, however, continue to be confounded by several factors such as variable lighting conditions, distance to the objects, and quality of images. Therefore, further development and evolution are required. Visual evaluation of wounds, the current gold standard, has served clinicians well but is clearly not the future of imaging in wound care.^6^

## 1 ULTRASOUND IMAGING

While the area measurements are essentially surface measurements, other technologies provide a more in‐depth insight into the wound and its underlying tissue. Advances in ultrasound technology have led to portable ultrasound solutions. Consequently, portable ultrasounds provide high‐quality imaging that was once limited to bulkier devices. Furthermore, ultrasound machines are becoming increasingly smaller, ranging from laptop‐based ultrasound machines to a variety of hand‐held scanners (e.g., Clarius^7^).

Portable ultrasounds can be used for 2D, 3D, or 4D imaging and are powered with advanced imaging capabilities for high‐contrast resolution and image optimisation.

Visualisation of tissue 3D structures up to a depth of several inches is possible making ultrasound imaging a powerful tool to study full‐thickness wounds. The portability of today's handheld devices makes it practical for use in wound care settings. Cloud storage and artificial intelligence analysis make this diagnostic approach more readily suited for the expert/non‐expert wound care world.

## 2 PERFUSION IMAGING

Wound physiology is complex, with the assessment and diagnosis of wounds being difficult. Vascular assessment is a critical in wound care to assess tissue health status and healability. Adequate tissue perfusion is recognised as a predictor of wound healing. Tissue oxygen saturation is a surrogate marker of wound perfusion. Ankle brachial index and transcutaneous oxygen measurement are the two clinically common methods used to measure vascular supply of wound tissue. However, both only provide a point measurement and are less suitable to assess the perfusion status over the entire wound area. Non‐invasive vascular assessments provide an adequate screening test, but their output is often limited and requires significant clinical interpretation.

New emerging sophisticated imaging technologies (e.g., MIMOSA Pro^8^) allow clinicians to obtain more information about the wounds they are treating and enable a better understanding of tissue microenvironments. These new imaging devices can assess tissue oxygenation, microvasculature, tissue health status, and wound microbiome. Use of these new diagnostic approaches will aid clinicians in making more informed treatment decisions.

Near‐infrared spectroscopy (NIRS) is an emerging imaging technology that can be used to evaluate functional tissue health status in the management of chronic wounds. This non‐contact device is hand‐held, mobile, and offers repeatable immediate images that can be used to determine site‐specific quantifiable levels of tissue oxygenation in and around the wound. This diagnostic tool uses differing optical signals based on the proportion of oxygenated haemoglobin found within the tissue capillary bed. The images obtained provide sufficient information for clinical assessment of tissue health. Near‐infrared imaging modalities can serve as an additional diagnostic assessment of wounds in which adequate perfusion is a concern. Correct interpretation of near‐infrared images obtained is critical.

## 3 FLUORESCENCE IMAGING

Increased wound bacteria loads can hinder healing and cause infection. Bacteria and biofilm may persist in the wounds even with good wound care practices. Elevated levels of tissue bioburden can further prolong wound chronicity. Although swabs and wound biopsies can be used to confirm presence of bacterial burden in wounds, but their use varies widely and results are often prolonged.

An essential component of wound care is debridement of devitalised or bacteria‐laden tissue that acts as a barrier to healing and can prevent the effectiveness of topical antimicrobial agents. Determining how much debridement is needed can be a challenge and can be over aggressive to ensure total removal. The immediate information on the presence and location of bacteria was essentially a guessing game until the development of fluorescence imaging. This technology shows bacterial‐loading of tissues providing a target for debridement. The immediate diagnostic information and feedback on treatment efficacy clinicians receive through the fluorescence imaging of bacteria is useful. Fluorescence imaging helps clinicians to understand the infection status of wounds and helps to monitor the effectiveness of clinical interventions.

Handheld fluorescence imaging devices (e.g., MolecuLight i:X^9^), are easy to use, non‐invasive and portable. This technology visualises potentially harmful bacteria on the wound surface and surrounding tissues not otherwise visible with the naked eye. The device emits a violet light that illuminates the wound and surrounding area, exciting the wound tissues and bacteria and resulting in fluorescence signals. The signals produced are tissue specific: endogenous tissue components such as collagen will fluoresce green, while pathologic bacteria fluoresce red, and pseudomonas will fluoresce cyan. The information captured in the images can aid in more targeted and thorough wound debridement, support clinical decisions and dressing selection, and aid in determining the need for antimicrobial therapy to improve clinical outcomes.

## 4 THERMAL IMAGING

Thermography provides significant diagnostic value and prognostic insight to a range of clinical problems. Thermal imaging devices (e.g., FLIR^10^) can potentially be used to provide information on systemic or local temperature abnormalities in tissue caused by ischemia, inflammation, or infection prior to clinical manifestations. The technique consists of comparing images obtained on both limbs and performing an asymmetrical analysis by subtracting mean temperature of the nonulcerated limb from the corresponding value of the ulcerated one. Long‐wave infrared thermography can measure radiant heat from a body surface and has been accepted as a valuable adjunct to standard investigations in the early detection of inflammation and infection. Research has shown that a temperature difference between a chronically infected wound and normal tissue has a specific elevated thermal gradient range of 3°C to 4°C.

The utility of thermography has been investigated in many clinical applications. The clinical utility can be split into two primary scenarios:

### 4.1 Inflammation based (Increased flow)

- Infection–powerful predictor of early Surgical Site Infection (SSI).
- Infection–adjunctive predictor of Diabetic Foot Infection (DFI).
- Autoimmune–Charcot Foot (diabetes).
- Autoimmune–Hidradenitis Suppurativa, Lupus Erythematosus, Calciphylaxis, etc.

### 4.2 Perfusion based (Decreased flow)

- Ischemic Diabetic Foot Ulcer (DFU).
- Surgical Free and Rotational Flaps.
- Angioplasty Surveillance.
- Pressure‐Induced including Deep Tissue Injury.
- Trauma‐Induced.

## 5 EMERGING LANDSCAPE

Current and emerging imaging technologies offer a deeper insight into both the wound and its underlying physiology. By visualising and obtaining this deeper insight the information provided offers higher clinical relevance of the assessment. While being in the early stages of implementation within wound care many have gained experience and relevance in other clinical and non‐clinical areas. The major benefit for wound care is as they become more and more integrated into wound care practice, they are fast becoming part of routine clinical workflow. This is especially true of the more portable and easier to use devices. While the interpretation of diagnostic output maybe be more complex this is often simplified via artificial intelligence and machine learning to present a diagnostic output.

The use of new diagnostic imaging technologies has started to change the clinical management of all types of wounds. Specifically, it has improved both the assessment and management of wounds and provides visual documentation that enhances patient engagement and facilitates better treatment compliance.
